# Effect of Extremely Low Frequency Electromagnetic Field and GABA_A_ Receptors on Serum Testosterone Level of Male Rats

**DOI:** 10.5812/ijem.11029

**Published:** 2013-10-21

**Authors:** Mahnaz Taherianfard, Aminolah Bahaddini, Sara Keshtkar, Mehdi Fazeli, Tahora Shomali

**Affiliations:** 1 Department of Physiology, School of Vetetrinary Medicine, Shiraz University, Shiraz, IR Iran; 2 Department of Biology, Faculty of Basic Science, Shiraz University, Shiraz, IR Iran; 3 Department of Biology, University of Scientific-Practical, Shiraz University, Shiraz, IR Iran; 4 Department of Pharmacology, School of Vetetrinary Medicine, Shiraz University, Shiraz, IR Iran

**Keywords:** Extremely Low Frequency Electromagnetic Fields, Muscimol Hydrobromide, bicuculline, Testosterone, Rat

## Abstract

**Background:**

GABA can influence the steroidogenesis in peripheral and central nervoussystems.

**Objectives:**

The present study investigates the interactive effect of GABA_A_ receptors and extremely low frequency electromagnetic field on serum testosterone level of male rats.

**Patients and Methods:**

Fifty adult male rats were randomly assigned into 10 groups. Groups 2, 4, 6, 8, and 10 were exposed to ELF-EMF for 30 days 8hrs per day; while, the remaining groups (1, 3, 5, 7, and 9) were sham exposed animals. At the end of the experiment, animals in groups 1 and 2 received normal saline; while, animals in groups 3 and 4 were treated with 1 mg/kg of bicuculline methiodide, and for animals of groups 5 and 6,3 mg/kg of bicuculline was injected. Animals of groups 7 and 8 were treated with 0.5 mg/kg of muscimol hydrobromide and rats in groups 9 and 10 received 2 mg/kg muscimol hydrobromide. About forty minutes after the injection, blood samples were collected and serum testosterone level was assayed using RIA.

**Results:**

Administration of muscimol hydrobromide at both doses to sham exposed rats significantly decreased serum testosterone level as compared to sham exposed animals which received saline. Administration of bicuculline methiodide without exposure to ELF-EMF, had no significant effect on testosterone level as compared to group 1. Serum testosterone levels of rats in different groups, exposed to ELF-EMF were statistically the same. Moreover, serum testosterone of exposed and sham exposed rats in each treatment showed no significant difference.

**Conclusions:**

No interactivity is present in modulatory effects of GABA_A_ receptors and ELF-EMFs on serum testosterone of male rats.

## 1. Background

Testosterone is the major male sex hormone which is primarily synthesized by Leydig cells of the testes and plays a critical role in the function and development of male reproductive system. Gamma-amino butyric acid (GABA) is well known as an important inhibitory neurotransmitter in the vertebrate central nervous system; however, it has been demonstrated that some endocrine organs such as somatotrophs of the anterior pituitary (1-3) and pancreatic islet cells (4-6) can also locally synthesize GABA and GABA receptors are present in these areas. Ritta et al. evaluated the effect of GABA on "in vitro" androgen production by rat testes and observed that the highest concentration of GABA was able to modify the basal and hCG-stimulated androgen production from adult and pubertal testes (7). A local GABAergic system has been recognized in adult Leydig cells in rodent and human testes (8). Naumenko et al. demonstrated that excitation of GABA_A_ receptors leads to the suppression, while excitation of the GABA_B_ receptors leads to the intensification, of the compensatory rise in the peripheral blood level of testosterone, after its decrease induced by hemi castration (9). 

Extremely low frequency electromagnetic fields (ELF-EMFs) are present wherever electric power is used. They are emitted by power lines, televisions, hair driers, cellular phones, etc (10). The possible health effects of ELF-EMFs on reproduction have been extensively studied; however, the results are often inconsistent and contradictory (11-14). These controversies are present when the testosterone levels are assayed in animals exposed to ELF-EMFs; while some authors observed no significant change in testosterone levels in rodents (14-16); others have reported an appreciable decrease of this hormone level when rats (17, 18) or guinea pigs (19) were exposed to these magnetic fields. Interactive effects of ELF-EMFs and GABA_A_ receptors for modulating the testosterone producing function of testicular Leydig cells have not been clarified yet. 

## 2. Objectives

Since the peripheral GABAergic system can modulate steroidogenesis in testis, and EMF exposure can modulate testosterone synthesis in testis; so the aim of the present investigation was to study the interactive effect of GABA_A_ receptors and ELF-EMF (50 Hz, 0.5 mT) on serum testosterone level of male rats.

## 3. Patients and Methods

### 3.1. Animals and Experimental Design

Fifty adult male Sprague-Dawley rats with a mean body weight of 200g were randomly assigned into 10 experimental groups (n=5 each). Groups 2, 4, 6, 8, and 10 were exposed to 50 Hz, 0.5 mT ELF-EMF for 30 days 8 hrs per day while the remaining groups (1,3,5,7,9) were sham exposed animals. At the end of this period, animals in groups 1 and 2 received normal saline; while, animals in groups 3 and 4 were treated with 1 mg/kg (low dose) of bicuculline methiodide (Sigma), and for animals of groups 5 and 6 3 mg/kg (high dose) of bicuculline methiodide was injected. Animals of groups 7 and 8 were treated with 0.5 mg/kg (low dose) of muscimol hydrobromide (sigma) and rats in groups 9 and 10 received 2 mg/kg (high dose) muscimol hydrobromide (20, 21). All these treatments were performed by IP injections and the volume of injection was kept equal for rats with same weights. 

During the experiment animals had free access to commercial pellets and tap water. The environmental conditions included 12 hrs light/12hrs dark cycles and temperature of about 22ºC. All procedures performed in this study were in accordance with the institutional guidelines of School of Veterinary Medicine, Shiraz University for using laboratory animals in scientific experiments. 

### 3.2. The Exposure System 

The magnetic field chamber used in the present study consisted of a 70×120 cm wooden cage with 30 cm height. Three coils of electrically insulated 1mm copper wire with 200 turns each were wound around the outer surface at equal distance.The coils were connected in parallel and sealed with adhesive bandage. The electrical source was an autotransformer with the input of 50 Hz and 220 V (22). The magnetic field inside the chamber was measured at different locations using a hand held Gauss/Tesla Meter. The field was homogenous in a zone with 21 cm distance from the transverse borders and 9 cm from the longitudinal borders. Cages were located inside this zone. The strength of the ELF-EMF was 0.5 mT in the homogenous zone.

### 3.3. Testosterone Assay

About forty minutes after the injection, animals were anesthetized by chloroform, and blood samples were collected by cardiocentesis. Serum testosterone level was assayed using an RIA kit (Immunotech, France).

### 3.4. Data Analysis

All data was presented as mean±SEM. For multiple comparisons among different groups, one-way ANOVA method, and Tukey's multiple comparison test as the post hoc were used. For testing between-subjects effects, factorial ANOVA analysis of variance was performed. P <0.05 was considered as the significance level.

## 4. Results 

### 4.1. Comparisons Among Different Groups

**Figure 1.**
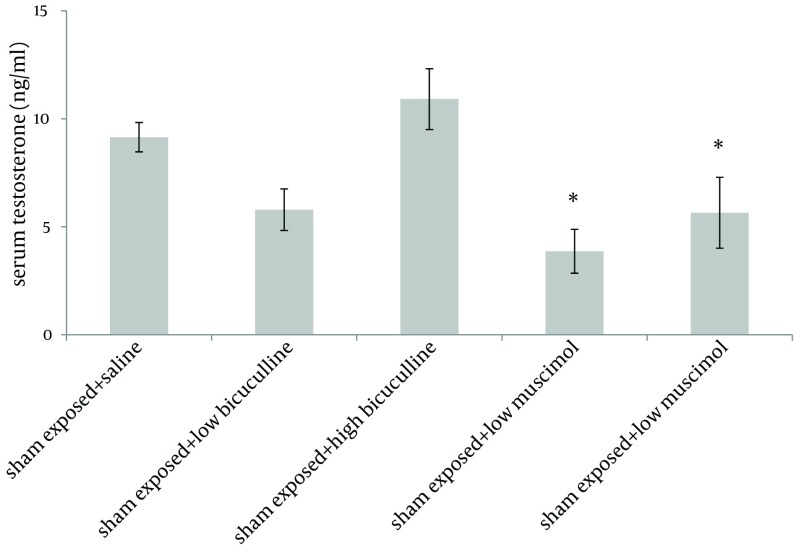
Serum Testosterone Level of Sham Exposed Rats (Mean and SEM) in Different Groups. *The asterisk demonstrates significant difference with sham exposed + saline group (P < 0.05).

**Figure 2.**
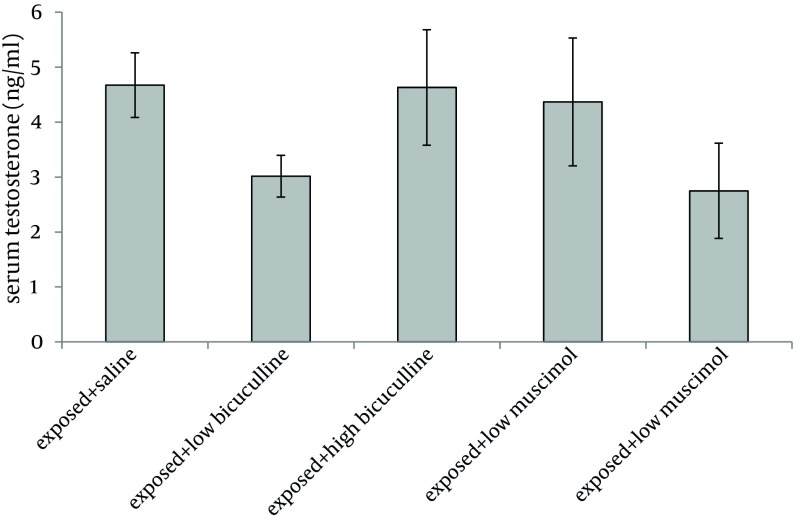
Serum Testosterone Level of Exposed Rats (Mean and SEM) in Different Groups No significant difference was observed among groups (P > 0.05).

**Figure 3.**
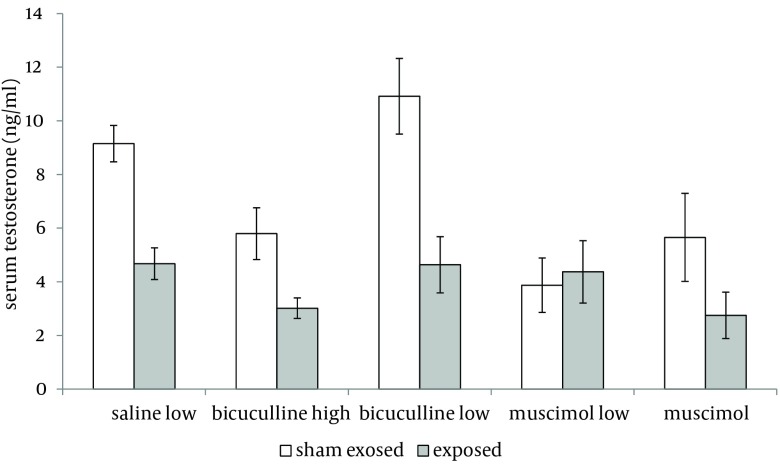
Serum Testosterone Level of Exposed and Sham Exposed Rats (Mean and SEM) With Different Treatments (Saline, Low Bicuculline, High Bicuculline, Low Muscimol Hydrobromide,and High Muscimol Hydrobromide). No significant difference was observed in each treatment group (P > 0.05).

Administration of muscimol hydrobromideat both low and high doses to sham exposed rats significantly decreased serum testosterone level as compared to sham exposed animals which received saline (P = 0.012 and P = 0.032 respectively). Although a slight increase was observed in rats treated with high dose bicuculline, administration of this agent without exposure to ELF-EMF, had no significant effect on testosterone level as compared to group 1 (Figure 1). Serum testosterone levels of rats in different groups, exposed to ELF-EMF were statistically the same. Results are summarized in Figure 2. Serum testosterone level of rats in group 2 (exposed to ELF-EMF and treated with saline) was only slightly lower than sham exposed animals which received saline (group 1). Moreover, serum testosterone of exposed and sham exposed rats in each drug treatment (bicuculline methiodideor muscimol hydrobromide) showed no significant difference (P > 0.05) (Figure 3). 

### 4.2. Between-Subjects Effects

No interactive effects were observed in drug×ELF-EMF, dose×ELF-EMF or drug×dose×ELF-EMF (P >0.05). 

## 5. Discussion

In the present study, the plausible interaction between GABA_A_ receptors function and ELF-EMF on serum testosterone levels of rats has been investigated.

GABA may affect testosterone level by both peripheral and central pathways. Adult Leydig cells possess GABA synthetic enzyme, as well as GABA_A_ and GABA_B_ receptors, which indicates that GABA may have a role in regulation of the functions of Leydig cells including testosterone production (8). Amikishieva et al. demonstrated that in unilateral hemi castrated rats, the maximal contribution of GABAergic mechanisms in the testosterone feedback regulation involves the GABA effect via the central GABA_A_receptors of the medio basal hypothalamus' serotoninergic neurons. Consistent with these findings, we observed that intraperitoneal administration of muscimol hydrobromide as a GABA_A_ receptor agonist significantly reduced testosterone level. Bicuculline methiodidedid not appreciably affect testosterone level in our study, although a slight increase was observed when high dose of bicuculline was administered (23). Taherianfard and Ahamdi reported that peripheral administration of bicuculline to rats only at a very high dose (3 mg/kg) is able to lower serum testosterone level; this may describe the absence of an appreciable response to bicuculline methiodidein our study (24). 

In the present study, exposure of animals to 50 Hz, 0.5 mT ELF-EMF for 30 days had no appreciable effect on testosterone level as compared to sham exposed animals, although a slight decrease was observed. Al-Akhras et al. exposed rats to 50 Hz, 25 µT ELF-EMF for 18 weeks and observed a significant decrease in serum testosterone level of rats only after 6 and 12 weeks of exposure (17). Mostafa et al. assayed serum testosterone levels of rats exposed to 50 Hz, 10 mT ELF-EMF for a period of 1, 2 and 4 weeks. At the end of 1 week no significant change was observed in testosterone level;however, this parameter was decreased significantly at the end of the second week as compared to sham exposed rats. Interestingly a remarkable increase was observed in testosterone level at the end of 4th week as compared to the second week of exposure, although it was still significantly lower than the control group. It seems that the strength of the field and/or duration of exposure may play a role in the final outcome, and this explains the discrepancies observed in different studies. The exact mechanism by which ELF-EMFs may affect serum testosterone level has not been clarified yet (18). Some authors have reported a significant increase in serum LH level accompanied by a decrease in testosterone level of rats exposed to ELF-EMFs (17, 18). This indicates that the effect of these ELF-EMFs on testosterone level is more suspected to be peripherally rather than central inhibition of hypophyseal hormones release.

We observed no interactive effects in drug × ELF-EMF, dose × ELF-EMF or drug × dose × ELF-EMF in rats treated with agents acting on GABA_A_ receptors and exposed to ELF-EMF. This supports the speculation that both peripheral and central GABA_A_ receptors do not modulate the effect of ELF-EMF on testosterone level. 

In conclusion, no interactivity is present in modulatory effects of GABA_A_ receptors and ELF-EMFs on serum testosterone of male rats.
